# Portable Interactive Pulse Tactile Recorder and Player System

**DOI:** 10.3390/s21134339

**Published:** 2021-06-25

**Authors:** Tzu-Chieh Hsieh, Chien-Min Wu, Cheng-Chung Tsai, Wen-Chien Lo, Yu-Min Wang, Shana Smith

**Affiliations:** Department of Mechanical Engineering, National Taiwan University, Taipei 10617, Taiwan; d07522017@ntu.edu.tw (T.-C.H.); d99522031@ntu.edu.tw (C.-M.W.); r07522608@ntu.edu.tw (C.-C.T.); r08522625@ntu.edu.tw (W.-C.L.); r07522628@ntu.edu.tw (Y.-M.W.)

**Keywords:** artificial neural network, empirical mode decomposition, intrinsic mode functions, pulse tactile player, pulse tactile recorder

## Abstract

Pulse palpation is an effective method for diagnosing arterial diseases. However, most pulse measurement devices use preconfigured pressures to collect pulse signals, and most pulse tactile simulators can only display standard or predefined pulse waveforms. Here, a portable interactive human pulse measurement and reproduction system was developed that allows users to take arbitrary pulses and experience realistic simulated pulse tactile feedback in real time by using their natural pulse-taking behaviors. The system includes a pulse tactile recorder and a pulse tactile player. Pulse palpation forces and vibrations can be recorded and realistically replayed for later tactile exploration and examination. To retain subtle but vital pulse information, empirical mode decomposition was used to decompose pulse waveforms into several intrinsic mode functions. Artificial neural networks were then trained based on intrinsic mode functions to determine the relationship between the driving signals of the pulse tactile player and the resulting vibration waveforms. Experimental results indicate that the average normalized root mean square error and the average *R*-squared values between the reproduced and original pulses were 0.0654 and 0.958 respectively, which indicate that the system can reproduce high-fidelity pulse tactile vibrations.

## 1. Introduction

Human pulses often reveal a person’s health conditions. Health care personnel and rescuers frequently use pulse palpation first to detect patients’ vital signs. Pulse taking also plays a prominent role in traditional Chinese medicine (TCM). TCM physicians typically take pulses directly by placing their index, middle, and ring fingers at the Cun, Guan, and Chi points on a patient’s wrists, respectively, by using light (<0.9 N), moderate (0.9–1.5 N), or heavy (>1.50 N) forces. Based on pulse depth, rate, shape, and strength, physicians can perceive the patient’s health conditions [1,2,3]. Pulse depth describes the vertical position of a pulse. Pulse rate describes the number of pulses per unit time. Pulse shape describes the width and length of a pulse. Pulse strength describes the forcefulness of the pulse against the finger pressure. Accurate pulse taking often requires rich clinical experience. Because pulse palpation is largely based on the individual’s finger tactile perception, it might be affected by numerous subjective and objective factors. Therefore, training entry-level health care personnel or TCM physicians in pulse taking and reading is extremely challenging.

Computer-aided pulse analysis tools have recently been developed to identify relationships between pulse features and diseases [4,5,6,7,8]. However, the accuracy of an analytical result is often based on the accuracy of the pulse signal acquisition device. With advances in sensor technology, small and lightweight sensors have been developed and applied to pulse measurement, such as optical [9,10], strain-gauge [9,11,12], pressure [11,12,13,14,15,16], piezoelectric [17], piezoresistive [18,19], and polyvinylidene-fluoride (PVDF) [20,21] sensors. Most research has used one or more of these sensors to detect cardiovascular disease or blood pressure. Prior studies have used mechanical methods or preconfigured pressures to collect pulse signals. Users only needed to follow certain steps to operate the devices to record pulse signals. However, TCM physicians and health care personnel often take pulses by switching back and forth between different pressures. Prior pulse taking devices lacked direct tactile interactions between patients and physicians and thus differed from the general practice of health care personnel and TCM physicians. Therefore, a more interactive pulse-taking system allowing users to take pulses by using their natural pulse-taking behaviors and preferred palpation pressures is needed.

In contrast to pulse measurement devices, relatively few studies on pulse tactile display have been conducted. Because of blood pressure against the vascular wall and the contraction and relaxation of heart muscles, a typical pulse waveform is composed of a percussion wave caused by early systolic pulse pressure, a tidal wave caused by late systolic pulse pressure, and a dicrotic notch caused by the rebound of blood [8,15,20]. Therefore, an effective pulse tactile display should present similar pulse features. The most common pulsation simulators are liquid based. For instance, the ViVitro Endovascular Simulator from ViVitro Labs uses pumps to deliver pulsatile flow. Lee et al. [1] developed a cardiovascular simulator using a stepping motor, slider-crank mechanism, piston pump, water, and glycerin to generate pulsatile flow for simulating typical TCM pulse waveforms. Koo et al. [22] built a radial pulsation simulator using a peristaltic pump and magnetorheological (MR) fluids. By controlling the viscosity and motion of the MR fluids with user-defined magnetic fields, arbitrary pulse waveforms were generated. Yang et al. [23] built a radial pulsation simulator using stepping motors and pistons to generate radial artery pressure waveforms reflecting the physiological characteristics of a human cardiovascular system. By carefully controlling the inflow of the fluid, the liquid-driven pulsation simulator created various standard pulse waveforms based on the cardiovascular conditions.

In addition to liquid-driven simulators, researchers have built pneumatic-driven pulsation simulators. A pneumatic-driven mechanism could avoid the problems of liquid-driven pulsation simulators, for example, sporadic pressure waves, bubbles, and leakage. For example, Santos-Carreras et al. [24] used five pneumatic balloon actuators to generate a pulse-like tactile sensation for teleoperated surgery. Although their device conveyed tactile information, detailed pulse features were ignored. Yang et al. [25] built a pneumatic-driven radial pulsation simulator based on a cam-follower mechanism controlled by a DC motor. However, cams needed to be fabricated to simulate different waveforms.

Although most liquid- or pneumatic-driven pulsation simulators can generate a favorable pulse tactile sensation, most are expensive, complex, and bulky. They can only simulate standard or predefined pulse waveforms, and real-time reproduction of arbitrary pulse waveforms was not possible. This study developed a portable and interactive pulsation system that can record and reproduce arbitrary pulse tactile information of any individual in real time. The pulsation measurement part of the system is referred to as the “pulse tactile recorder”, and the pulsation reproduction part is named the “pulse tactile player”. The pulse tactile recorder allows users to take pulses by using their natural pulse-taking behaviors and preferred palpation pressures. The pulse tactile player can realistically reproduce the tactile vibration in real time for further diagnosis or training.

## 2. Pulse Tactile Recorder

### 2.1. Hardware Design

**Figure 1:**
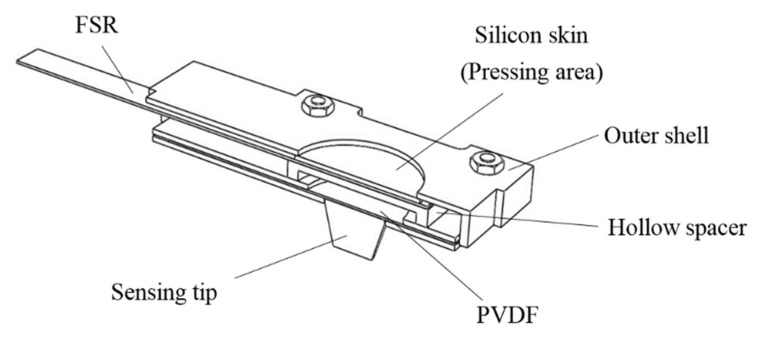
Section view of the pulse tactile recorder.

Because pulse tactile vibrations vary depending on palpation forces, in addition to vibration information, palpation forces should also be recorded. The structure of the pulse tactile recorder includes a vibration sensor, a force sensor, a sensing tip, a hollow spacer, and a silicon skin. Figure 1 presents a section view of the structure. The PVDF piezo film vibration sensor SDT1-028K (Measurement Specialties) was selected to detect pulse vibration signals because the sensor performs well at both low and high frequencies and the resonant frequency points are >10 MHz. The dimensions of the sensor are 16 mm × 41 mm × 75 μm. The output sensitivity is 15–20 mV/μ strain. The polymer film force sensor FSR 402 (Interlink Electronics) was selected to measure pressing force. The force sensor is 18.28 mm in diameter and 0.45 mm in thickness. The force sensitivity range is between 0.2 and 20 N, with analog output.

On the top of the force sensor is a round pressing area, covered with a silicon skin, where users can place their fingers and exert a pressing force. A hollow spacer is placed between the force and vibration sensors so that the vibration sensor can freely vibrate without being suppressed by the pressing force from fingers. A conical tip, which is used to contact the patients to detect pulse signals, is attached to one side of the vibration sensor.

The Taguchi method was used to optimize the structure design. Table 1 shows four important factors (A: height of the sensing tip; B: thickness of the hollow spacer between FSR and PVDF; C: number of clamping sides; D: material of the sensing tip) and the corresponding levels considered in the structure design. An L_9_ orthogonal array was used to design the experiment. Table 2 shows the level chosen for each factor in each experiment. Each design was evaluated using a cam mechanism described in the next section. Different hardware layouts might affect sensor sensitivity. If a design provides a higher voltage output, that means the design has higher sensitivity towards deformation. In addition, an effective recorder’s measured profile should fit the original waveform profile as much as possible. Therefore, in this study, a score was given using the following equation to evaluate a design:

*Score* = *R-squared value* + *norm peak volt* (1)

where “*R-squared value*” is the fit between the original cam profile and the measured profile and “*norm peak volt*” is the normalized peak voltage of the output signals of each design. Based on the results of the 9 experiments, a standard analysis of the Taguchi method was conducted. The analysis results suggest that factor A with level 1 (5 mm of sensing tip), factor B with level 3 (2 mm of spacer thickness), factor C with level 2 (4 clamping sides), and factor D with level 1 (PLA sensing tip) formed the optimum structure design. The suggested design happened to be one of the 9 experiments in Table 2, with a score of 1.97. In order to check if longer sensing tips led to higher scores, a sensing tip with 7 mm was tested. The score of the 7 mm tip was 1.81 (*R*^2^ = 0.96 and *norm peak volt* = 0.85), which is lower than that of the 5 mm.

**Figure 2:**
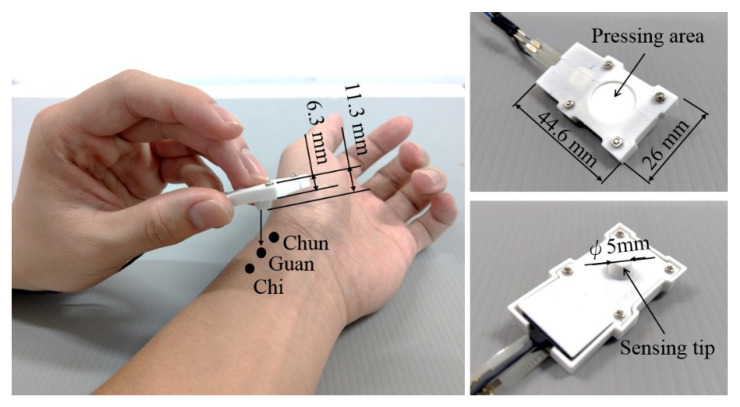
Use of the pulse tactile recorder to take pulses at the Cun, Guan, and Chi points (**left**). Top (**upper right**) and bottom (**lower right**) views of the recorder.

The vibration sensor, force sensor, hollow spacer, and silicon skin are clamped inside a shell with total dimensions of 44.6 × 26 × 6 mm^3^. The whole tactile recorder weighs only 10 g. Users can interact with patients using their natural pulse-taking behaviors with their preferred palpation forces when they are taking pulses, as illustrated in Figure 2.

### 2.2. Pulse Signal Verification

**Figure 3:**
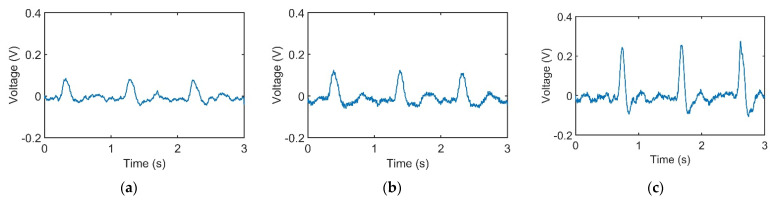
Pulse signals with (**a**) light (0.78 N); (**b**) moderate (1.18 N); and (**c**) heavy (1.76 N) pressure at the Guan point on the left wrist.

Figure 3a–c represents pulses taken using the pulse tactile recorder with three pressing forces of 0.78, 1.18, and 1.76 N, respectively. The pulse tactile recorder can record miniscule pulse signals; however, it is apparent that the signals in Figure 3 lack typical pulse features described in [8,15,20]. A typical pulse waveform is composed of a percussion wave, a tidal wave, and a dicrotic notch caused by the rebound of blood. All the features must lie above a baseline. However, the signals in Figure 3 fall below the baseline (0 V) after the peak signals. The reason for this was that when pressure was applied to the PVDF piezo film vibration sensor, the PVDF deformed and generated charges proportionate to the pressure applied. However, the oscilloscope displayed only the voltage generated by the charges. Therefore, the displacement of the PVDF was actually proportional to the integral of the voltage (charge accumulation). An experiment using a cam profile was then performed to verify the accuracy of the recorder, as described as follows.

**Figure 4:**
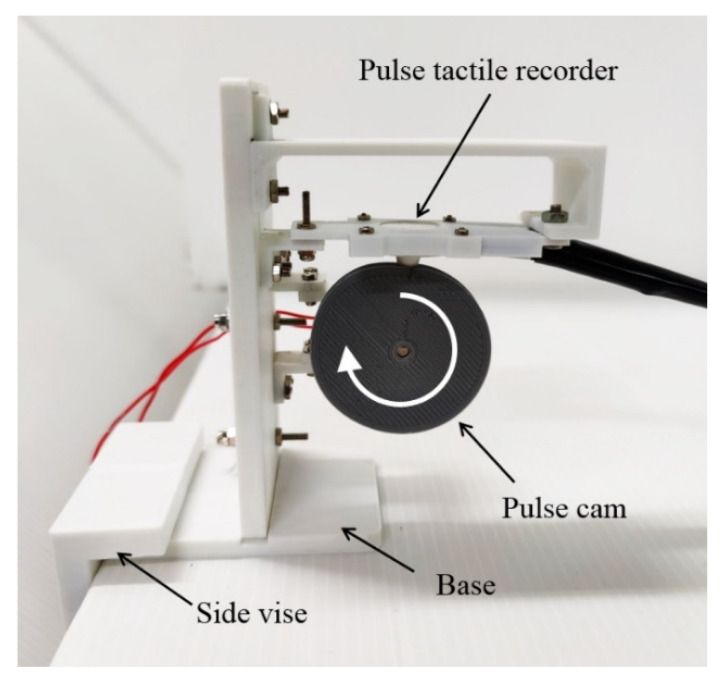
Pulse verification mechanism.

A cam profile was designed according to a period of pulse waveform using SolidWorks Motion Analysis. The cam was driven by a DC motor at a constant rotational speed of 60 rpm. The pulse tactile recorder was placed on top of the cam, and the sensing tip was contacted with the cam, as portrayed in Figure 4.

**Figure 5:**
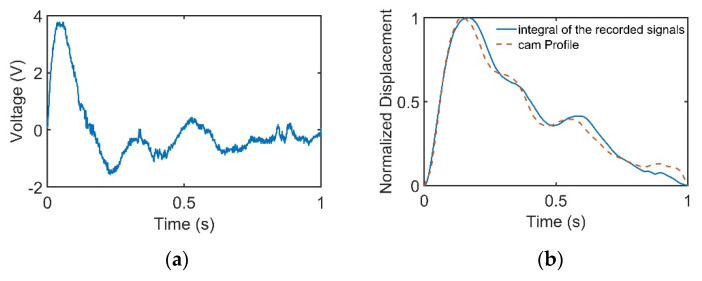
Cam profile is linear with the integral of the recorded signals. (**a**) Cam profile signals recorded by the pulse tactile recorder. (**b**) Comparison of the cam profile and the integral of the recorded signals.

When the cam rotated, the cam profile would deform the PVDF sensor according to the profile of the cam, which was a period of pulse waveform. The recorded PVDF signals are presented in Figure 5a. The signals were then integrated to find the displacement variation of the PVDF sensor, as provided in Figure 5b.

The experiment was repeated 10 times. The integral of the recorded signals and the original cam profile were compared using the normalized root mean square error (NRMSE) and *R*-squared values. The NRMSE was calculated as follows:

(2) $$NRMSE=\frac{RMSE}{P_{max}-P_{min}}$$

where *P_max_* and *P_min_* denote the maximum and minimum values of the original cam waveform. Table 3 shows that the average NRMSE and *R*-squared values were 0.046 and 0.983, respectively, with low standard deviations, which indicates that the pulse tactile recorder could measure pulses with high accuracy and repeatability.

**Figure 6:**
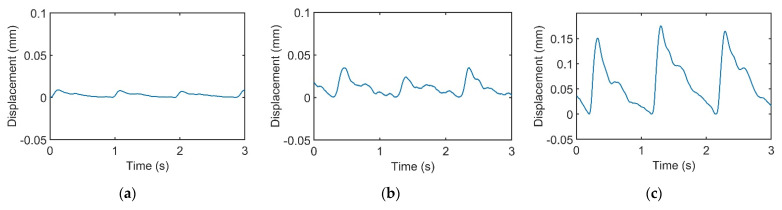
Integrals of the recorded pulse signals in Figure 3. Pulse waveforms of (**a**) light, (**b**) moderate, and (**c**) heavy forces.

In this study, the signals recorded by the recorder are called “pulse signals”, and the integrals of the pulse signals are called “pulse waveforms”. Figure 6 displays the pulse waveforms of the pulse signals in Figure 3. All the pulse waveforms now revealed relevant typical pulse features.

## 3. Pulse Tactile Player

Piezoelectric benders were selected as the actuators to develop the portable pulse tactile player because of their wide bandwidth, fast response, small size, light weight, and low cost. Piezoelectric bimorph benders from Unictron Technologies were used. The active layer of the bimorph is made with lead zirconate titanate ceramic with 0.19 mm thickness for each side. The passive layer is stainless steel with 0.1 mm thickness. The total dimensions of the player are 60 × 20 × 0.48 mm^3^, and the driving voltage is ±48 V DC. The force generated by one bender is approximately 0.5 N, and the maximum displacement is approximately 1 mm. To generate sufficient pulse force, five piezoelectric benders were used.

**Figure 7:**
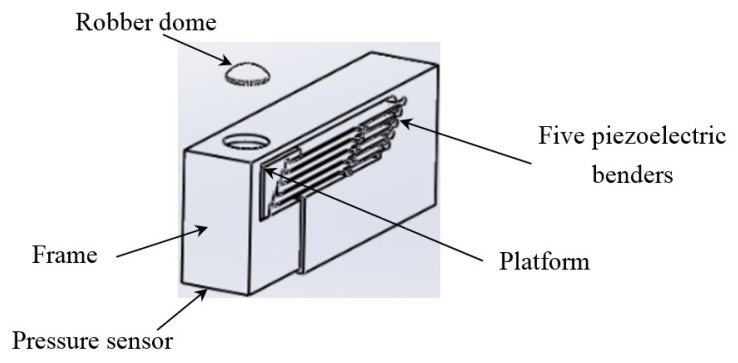
Actuating part of the pulse tactile player.

**Figure 8:**
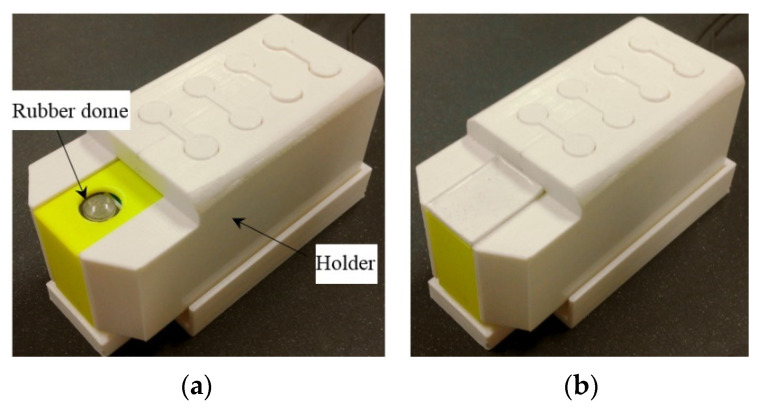
Complete unit of the pulse tactile player. (**a**) Without a silicon skin. (**b**) With a silicon skin.

The actuating part of the pulse tactile player is pictured in Figure 7. One end of the bender is fixed to a frame, and the other end of the bender is attached to a movable platform. The top of the frame has a 10 mm diameter round hole with a rubber dome placed in the center. The force sensor FSR 402 is placed under the frame to detect finger force. The actuating part is enclosed inside a holder to form a complete unit, as depicted in Figure 8a. Because the sensation of human skin also affects tactile perception, a silicon skin with shore hardness A-10 was fabricated and placed on top of the robber dome, as portrayed in Figure 8b. The final dimensions of the pulse tactile player are 115 × 56.5 × 58 mm^3^, and the total weight is 310 g. The device is lightweight and portable compared to other pulse tactile displays.

**Figure 9:**
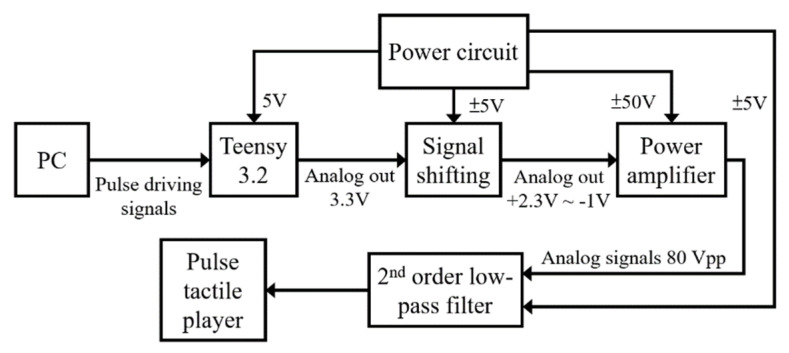
System block diagram of the control unit of the pulse tactile player.

The system block diagram of the control unit is displayed in Figure 9. A Teensy 3.2 USB development board is used to process the driving signals of the pulse tactile player. The amplifier OPA454 (Texas Instruments) is used to amplify the driving signals from 3.3 to 80 Vpp to drive the pulse tactile player.

## 4. Linear Model

In theory, a linear relationship between the driving signals and the displacement of the piezoelectric benders should exist. Therefore, in most early research, a linear model was adopted to drive a piezoelectric actuator [26,27]. However, prior research also showed that nonlinear relationships might exist due to hysteresis [28,29]. Since pulse waveforms contain many important but minuscule features [30], in the present study, it was not clear if a linear model was sufficient to describe the relationship between the driving signals and the displacements of the piezoelectric benders when simulating a human pulse. In addition, finger pressures might suppress the vibration of the benders and cause a nonlinear relationship. In order to test the linearity of the pulse tactile player, two experiments were conducted. First, a linear relationship was assumed between the driving voltage and the output waveforms. Then, a nonlinear relationship was assumed. The results of the two experiments were compared using the NRMSE and *R*-squared values between the reproduced and original pulses.

Eight sets of 8 sec wrist pulse signals were collected in each of the three force ranges (<0.9, 0.9–1.5, and >1.5 N) from the pulse tactile recorder. According to the Nyquist–Shannon sampling theorem [31], the sampling rate should be at least two times larger than the target frequency. The sampling rate of the pulse tactile recorder is 1 kHz, which is much higher than the frequency of the human pulse (0.6–2 Hz) so that it can contain sufficient critical pulse information [32]. A mean filter was used to down sample the data to 250 Hz to remove noise, resulting in 2000 data points in each dataset. The pulse signals were then integrated to obtain the original pulse waveforms.

**Figure 10:**
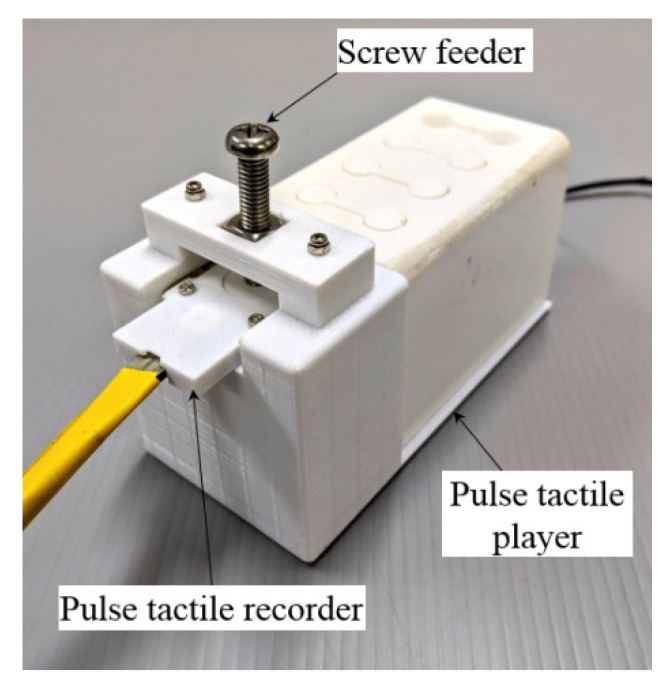
Data acquisition and verification mechanism.

A verification setup was designed to verify the output waveforms (Figure 10). A screw feeder was adjusted to provide the same amount of force on the pulse tactile recorder as the recorded original. A simple linear model was used to drive the pulse tactile player based on the original pulse waveforms. The pulse tactile recorder was placed on the silicon skin of the pulse tactile player to record the vibration signals. Finally, the integrals of the output signals were compared with the original pulse waveforms using the NRMSE and *R*-squared values. Table 4 and Table 5 show that the average NRMSE and the average *R*-squared values between the original pulse waveforms and the output waveforms (integral of the output signals) were 0.572 and 0.915, respectively.

## 5. Nonlinear Model

### 5.1. Artificial Neural Network

In this study, neural networks were used to model the nonlinear relationship between the driving signals and the output waveforms of the pulse tactile player [33]. A back-propagation multilayer perceptron artificial neural network (ANN) was used to train a nonlinear function, *T_NN_*. The training data for the ANN included input data *P*, which was the displacement of the pulse tactile player, and output data *U*, which was the driving voltage of the pulse tactile player, as described in (3).

(3) $$T_{NN}\left(P\right)=U$$

After the ANN model was well trained, the corresponding driving voltage to drive the pulse tactile player to reproduce a given pulse tactile vibration could be identified.

### 5.2. ANN Training Data Collection

The same data acquisition mechanism was used to collect the training data (Figure 10). The pulse tactile recorder was placed on top of the silicon skin of the pulse tactile player. A screw feeder was used to provide specified forces. The driving voltage of the pulse tactile recorder would be the output data *U* of the ANN model, and the integral of the recorded signals from the pulse tactile player would be the input data *P* for the ANN model.

Another ten sets of 8 s wrist pulse signals were collected in each of the three force ranges (<0.9, 0.9–1.5, and >1.5N) from the pulse tactile recorder. In all, 30 sets of pulse signals were collected. A mean filter was used to down sample the data to 250 Hz to remove noise, resulting in 2000 data points in each dataset. The pulse signals were then integrated to obtain the corresponding pulse waveforms.

**Figure 11:**
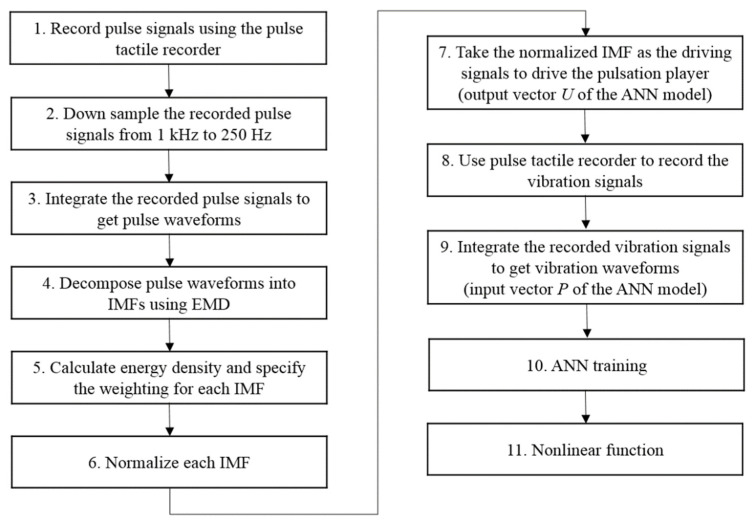
Flowchart of the ANN training data collection process.

However, if pulse waveforms were to be used to train the ANN model, some miniscule pulse information might be misidentified as noise and removed during the training. To retain both low- and high-frequency miniscule pulse information, empirical mode decomposition (EMD) was used to decompose pulse waveforms into several intrinsic mode functions (IMFs), which were then used to train the ANN model on various frequency scales to retain miniscule characteristics of the pulse waveforms while removing high-frequency noises at the same time. A flowchart of the training data obtainment is presented in Figure 11.

The EMD method was designed for analyzing nonlinear and nonstationary time series data [34,35]. EMD decomposes signals into several IMFs using a sifting process in which predetermined basis functions are not required. IMFs can represent the various frequency scales of the original signals. The combination of all IMFs can accurately represent the original signals without the loss of details during data conversion. EMD has been successfully applied to biological data analysis [36,37,38]. Prior research has shown that using the concept of “divide-and-conquer”, EMD-based neural networks could achieve higher training accuracy and efficiency [39,40]. Research has also demonstrated that EMD is more precise than the Fourier filter in extracting low-frequency components [41] and more efficient than wavelet transform in classifying disturbances [42].

In EMD, cubic splines are used to produce an upper envelope and a lower envelope of the signals. The mean of the two envelopes may be represented as $m_{1}\left(t\right)$. The difference between the original signal *x*(t) and $m_{1}\left(t\right)$ is the first component $h_{1}\left(t\right)$, as follows:

(4) $$h_{1}\left(t\right)=x\left(t\right)-m_{1}\left(t\right)$$

The same procedure is repeated to find the upper and lower envelopes of $h_{1}\left(t\right)$, and the mean value is represented as $m_{11}\left(t\right)$. $m_{11}\left(t\right)$ is then subtracted from $h_{1}\left(t\right)$ to obtain the second component $h_{11}\left(t\right)$, as follows:

(5) $$h_{11}\left(t\right)=h_{1}\left(t\right)-m_{11}\left(t\right)$$

The same sifting procedure is repeated *k* times until a stop criterion is satisfied:

(6) $$h_{1k}\left(t\right)=h_{1\left(k-1\right)}\left(t\right)-m_{1k}\left(t\right)$$

In this study, the Matlab EMD Toolbox and the Cauchy-type convergence criterion were used [43], as follows:

(7) $$SD_{k}=\frac{\sum_{t=0}^{T}|h_{1\left(k-1\right)}\left(t\right)-h_{1k}\left(t\right){|}^{2}}{\sum_{t=0}^{T}|h_{1\left(k-1\right)}\left(t\right){|}^{2}}$$

where *T* is the data length. The threshold was set to be 0.1. If *SD* was smaller than the threshold, the sifting process would stop. The resulting $h_{1k}\left(t\right)$ was an IMF and defined as $IMF_{1}\left(t\right)$. Each IMF satisfied the following two conditions: (1) the number of extrema and the number of zero crossings must either equal or differ at most by one, and (2) at any data point, the mean value of the envelope defined using the local maxima and the envelope defined using the local minima is zero [35].

$IMF_{1}\left(t\right)$ was then separated from x(t) as follows:

(8) $$r_{1}\left(t\right)=x\left(t\right)-IMF_{1}\left(t\right)$$

The residue, $r_{1}\left(t\right)$, was treated as a new data set and subjected to the same sifting process until the residue, $r_{n}\left(t\right)$, became a monotonic function from which no more IMFs could be extracted, as follows:

(9) $$r_{n}\left(t\right)=r_{n-1}\left(t\right)-IMF_{n}\left(t\right)$$

**Figure 12:**
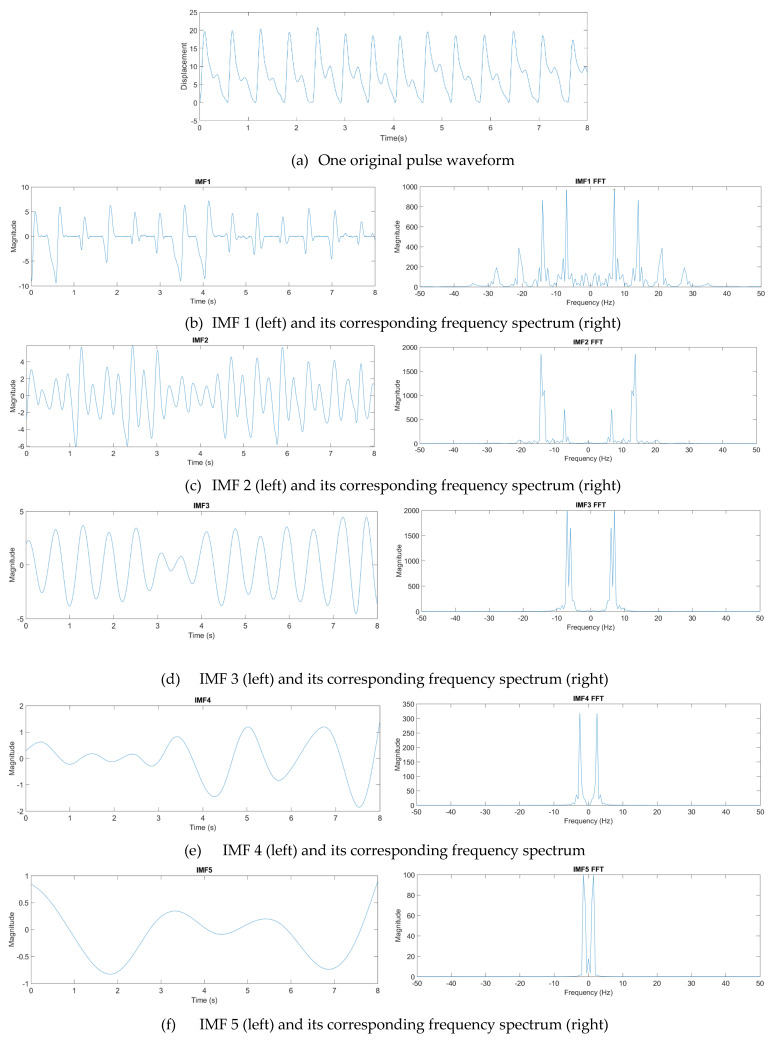

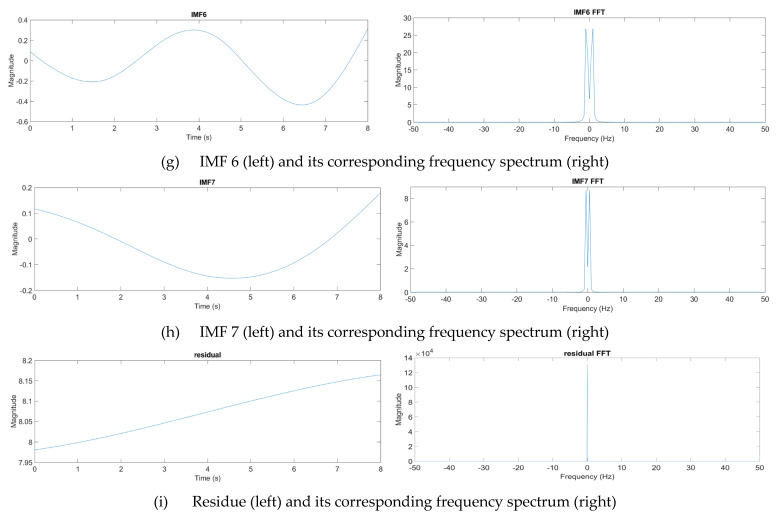
Decomposition of a pulse waveform into seven IMFs and one residue. (**a**) Pulse waveform. (**b**) IMF 1, (**c**) IMF 2, (**d**) IMF 3, (**e**) IMF 4, (**f**) IMF 5, (**g**) IMF 6, (**h**) IMF 7, and (**i**) residue.

The original signal *x*(*t*) could be expressed as follows:

(10) $$x\left(t\right)=\overset{n}{\underset{i=1}{\sum }}IMF_{i}\left(t\right)+r_{n}\left(t\right)$$

where *n* denotes the total number of IMF components. From experience, one eight-second pulse waveform could be decomposed into at most seven IMFs. Figure 12 presents one pulse waveform and its seven IMFs and one residue. Each IMF had its own main peak frequency and energy intensity. In this study, the energy intensity of each IMF was taken as the weighting of the IMF. Usually, the first three IMFs have the highest energy intensities and affect the waveform the most.

The IMFs were then normalized and taken as the driving signals to drive the pulse tactile player. The vibration signals were recorded using the pulse tactile recorder. Finally, the driving signals and the integrals of the recorded signals provided the output data and input data for the ANN training model, respectively. Because residue was the basic trend of the pulse waveform, it was not used in the training. Here, ten sets of pulse data, each containing 2000 data points, were collected in each force level, and each set of pulse data was decomposed into seven IMFs. Therefore, in total, 70 sets of ANN training data (or 140,000 data points) were provided for each force range.

### 5.3. ANN Training Model

For each force range, one ANN was trained. In this study, in order to process and reproduce each data point in real time, the input layer and output layer both comprised 1 neuron. In other words, both the input data *P* and output data *U* contained one data point only. However, the number of hidden layers, the number of neurons in the hidden layers, and the activation functions were obtained by a trial and error method until satisfactory accuracy and training time were achieved. Finally, one hidden layer comprising 10 neurons was adopted. The sigmoid function and hyperbolic tangent sigmoid function were selected as the activation functions for the hidden layer and the output layer, respectively, as described in (11) and (12).

Sigmoid function

(11) $$f\left(n\right)=\frac{1}{1+e^{-n}}$$

Hyperbolic tangent sigmoid function

(12) $$f\left(n\right)=\frac{2}{\left(1+e^{-2n}\right)}-1$$

In this study, the number of iterations for the ANN training was set to 10^4^ epochs. The learning rate of the training process was set to 0.8. Table 6 lists the NRMSEs of the training results.

### 5.4. Pulse Reproduction and Verification

#### 5.4.1. Driving Signals

**Figure 13:**
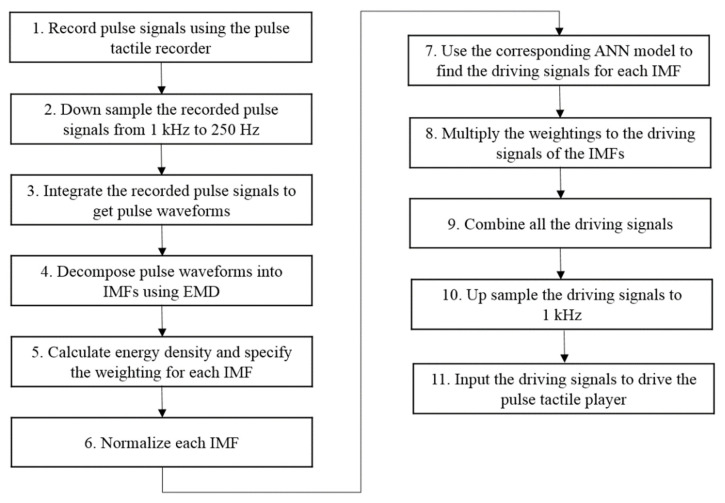
Pulse reproduction flowchart.

Figure 13 presents a flowchart for reproducing a complete pulsation. Steps 1 to 6 are represented in Figure 11. Based on the force range, Step 7 uses the corresponding ANN model to determine the driving signals for each IMF. Step 8 multiplies each driving signal with the corresponding weighting obtained in Step 5. Because a pulse waveform is decomposed into several IMFs, in order to reproduce a complete pulse vibration, all the driving signals of the IMFs must be combined, as described in Step 9. Finally, the driving signals are up sampled to 1 kHz to obtain the final driving signals of the pulse tactile player, reproducing a complete pulsation.

#### 5.4.2. Verification Results

The same eight sets of 8 sec wrist pulse signals used in the linear model were taken to verify the reproduction performance. The verification setup was identical to the data acquisition and verification mechanism in Figure 10. The integrals of the recorded signals were compared with the original pulse waveforms using the NRMSE and *R*-squared values.

As can be observed in Table 7, the average NRMSE of the differences between the reproduced pulses and the original pulses is 0.065. This indicates that the trained ANN models predict the pulse waveforms quite well. As Table 8 reports, the average *R*-squared value is 0.958, indicating that a high linear relationship between the reproduced and original pulse waveforms exists.

## 6. Discussion

In this study, a simple linear model and ANN models were used to describe the relationship between the driving signals of the pulse tactile player and the pulse tactile vibrations. In order to see if there were any significant differences between the linear model and ANN models, independent sample *t*-tests with the significant level of 0.05 were conducted. Table 9 shows that the NRMSEs of the ANN models of the three forces are all significantly lower than that of the linear model. It indicates that compared to the linear model, the ANN models have less noise, and the data points of the reproduced waveforms are closer to the original pulse waveforms.

Table 9 also shows that the *R*-squared values of the ANN models of the moderate and heavy forces are significantly higher than that of the linear model. Although the *R*-squared value of the ANN model of the light force is not significantly higher, the average value is still higher than that of the linear model. It indicates that the ANN models could provide better fit than the linear model.

Usually, pulse signals taken by light forces are indistinct and contain lots noise. Table 4 shows that the average NRMSE of the light forces is 0.93. To remove signal noise but retain subtle pulse information at various frequency scales, the EMD method was used to decompose pulse waveforms into several IMFs during the ANN training. Table 7 shows that using EMD and ANN models, the average NRMSE of the light forces reduces to 0.062.

By combining all the driving signals of the IMFs, the final driving signals for the pulse tactile player to reproduce a complete pulse tactile vibration were obtained. Figure 12 and Table 6 show that the first three IMFs contain relatively high energy intensities and low NRMSE values. Since the energy intensity of each IMF is used as the weight of the IMF when combining all the driving signals of the IMFs, the first three IMFs affect the final driving signals the most. Therefore, even most of the NRMSE values of IMFs 4–7 are high in Table 6, the average NRMSEs of the ANN models in Table 7 is still very low. The average NRMSE and *R*^2^ of the pulse player using the ANN models are 0.065 and 0.958, respectively. The results of the verification indicate that the system can reproduce high-fidelity pulse tactile vibrations.

This study selected a PVDF piezo film vibration sensor to detect pulse vibration signals. Different hardware layouts might affect the measurement performance. Taguchi method results show that the piezo film vibration sensor needs to be firmly clamped and the material of the sensing tip needs to be hard to record high fidelity pulse signals. The results show that the average NRMSE and *R*^2^ of the pulse recorder are 0.046 and 0.983, respectively, which indicate that the pulse tactile recorder could measure pulses with high accuracy and repeatability.

The focus of the present study was to design and objectively verify the pulsation recorder and player system. In the future, experienced TCM physicians and patients with different pulse features will be recruited to conduct a subjective clinical trial. In addition, other machine learning methods may be applied to further improve the reproduction performance of the pulse tactile player. A pulse tactile recorder and player system for multiple fingers will also be developed.

## 7. Conclusions

Human pulses contain much minuscule but important information. Trained TCM physicians and health care personnel are able to discern subtle pulse differences. Therefore, to realistically and accurately simulate human pulse tactile feedback, accurate pulsation measurement and display devices are needed. This study successfully developed a lightweight and portable pulse tactile recorder and player system. Users can interact with patients by using their natural pulse-taking behaviors and preferred palpation forces. After taking arbitrary pulses, the system can render the same pulse tactile feedback under the same palpation forces in real time. The system is expected to be used as a tool to train entry-level health care personnel and TCM physicians in taking and reading pulses.

## Figures and Tables

**Table 1:** Factors and levels used in the Taguchi method.

Factor		Level	
	1	2	3
Height of the sensing tip	5 mm	4 mm	3 mm
Thickness of the hollow spacer between FSR and PVDF	3 mm	1 mm	2 mm
Number of clamping sides	2	4	
Material of the sensing tip	PLA	Silicon	

**Table 2:** Taguchi method experiment results.

Experiment	Factor Level (ABCD)	*R* ^2^	Normalized Peak Voltage	Score
1	1111	0.98	0.65	1.63
2	1222	0.97	0.43	1.4
3	1321	0.97	1	1.97
4	2121	0.94	0.63	1.57
5	2211	0.91	0.57	1.48
6	2312	0.93	0.56	1.49
7	3122	0.92	0.49	1.41
8	3212	0.93	0.35	1.28
9	3321	0.95	0.83	1.78

**Table 3:** Comparison between the integral of the recorded signals and original cam waveform.

No.	NRMSE	*R* ^2^
1	0.049	0.984
2	0.051	0.983
3	0.052	0.983
4	0.051	0.984
5	0.051	0.983
6	0.039	0.984
7	0.042	0.983
8	0.042	0.982
9	0.042	0.983
10	0.042	0.983
Average	0.046	0.983
S.D.	0.005	0.001

**Table 4:** NRMSE of the linear model.

Subject No.	Force			Average
	Light	Moderate	Heavy	
1	0.785	0.669	0.140	0.531
2	0.787	0.630	0.141	0.519
3	1.066	0.723	0.106	0.632
4	1.152	0.642	0.107	0.634
5	1.257	0.667	0.108	0.677
6	0.771	0.667	0.103	0.514
7	0.797	0.652	0.125	0.525
8	0.827	0.697	0.119	0.548
Average	0.930	0.668	0.119	0.572
S.D.	0.196	0.030	0.015	0.065

**Table 5:** *R*-squared values of the linear model.

Subject No.	Force			Average
	Light	Moderate	Heavy	
1	0.942	0.913	0.833	0.896
2	0.941	0.908	0.955	0.935
3	0.897	0.928	0.911	0.912
4	0.940	0.922	0.929	0.930
5	0.897	0.921	0.953	0.924
6	0.944	0.923	0.885	0.917
7	0.927	0.837	0.955	0.907
8	0.882	0.909	0.910	0.900
Average	0.921	0.908	0.917	0.915
S.D.	0.025	0.029	0.042	0.014

**Table 6:** NRMSEs of the ANN training results.

IMF	NRMSE		
	Light	Moderate	Heavy
1	0.045	0.025	0.027
2	0.077	0.023	0.025
3	0.119	0.084	0.085
4	0.130	0.105	0.115
5	0.121	0.069	0.088
6	0.106	0.012	0.060
7	0.113	0.091	0.104

**Table 7:** NRMSE of the ANN models.

Subject No.	Force			Average
	Light	Moderate	Heavy	
1	0.100	0.085	0.067	0.084
2	0.047	0.064	0.063	0.058
3	0.054	0.082	0.058	0.064
4	0.067	0.055	0.070	0.064
5	0.048	0.065	0.052	0.055
6	0.069	0.054	0.060	0.061
7	0.057	0.066	0.066	0.063
8	0.059	0.087	0.077	0.074
Average	0.062	0.070	0.064	0.065
S.D.	0.017	0.013	0.008	0.009

**Table 8:** *R*-squared values of the ANN models.

Subject No.	Force			Average
	Light	Moderate	Heavy	
1	0.956	0.984	0.978	0.973
2	0.887	0.927	0.968	0.928
3	0.91	0.98	0.958	0.95
4	0.976	0.99	0.966	0.977
5	0.968	0.988	0.964	0.974
6	0.956	0.98	0.955	0.964
7	0.968	0.972	0.91	0.95
8	0.931	0.939	0.966	0.945
Average	0.944	0.97	0.958	0.958
S.D.	0.0317	0.0237	0.0206	0.0172

**Table 9:** *p*-values between the linear model and the ANN models.

	Pressure			
	Light	Moderate	Heavy	All
NRMSE	0.000	0.000	0.000	0.000
*R*-SQUARED	0.134	0.000	0.025	0.000

## Data Availability

Data is available upon request from the authors.
